# High frequency of CD8 escape mutations in elite controllers as new obstacle for HIV cure

**DOI:** 10.1080/21505594.2022.2129353

**Published:** 2022-10-03

**Authors:** María A. Navarrete-Muñoz, Ricardo Ramos, África Holguín, Alfonso Cabello, Miguel Górgolas, José M. Benito, Norma Rallón

**Affiliations:** a HIV and Viral Hepatitis Research Laboratory, Instituto de Investigación Sanitaria Fundación Jiménez Díaz, Universidad Autónoma de Madrid (IIS-FJD, UAM), Madrid, Spain; b Hospital Universitario Rey Juan Carlos, Móstoles, Spain; c Unidad de Genómica, Scientific Park of Madrid, Madrid, Spain; d HIV-1 Molecular Epidemiology Laboratory, Instituto Ramón y Cajal de Investigación Sanitaria(IRYCIS), Madrid, Spain; e Division of Infectious Diseases, Hospital Universitario Fundación Jiménez Díaz, Madrid, Spain

**Keywords:** HIV reservoir, resting memory CD4+ T-cells, elite controllers, CTL response, HIV-Gag proviral DNA, next generation sequencing (NGS)

## Abstract

Accumulation of mutations in epitopes of cytolytic-*T*-lymphocytes immune response (CTL) in HIV-reservoir seems to be one of the reasons for shock-and-kill strategy failure. Ten non-controller patients on successful cART (TX) and seven elite controllers (EC) were included. HIV-Gag gene from purified resting memory CD4+ *T*-cells was sequenced by Next-Generation-Sequencing. HLA class-I alleles were typed to predict optimal HIV-Gag CTL epitopes. For each subject, the frequency of mutated epitopes in the HIV-Gag gene, the proportion of them considered as CTL-escape variants as well as their effect on antigen recognition by HLA were assessed. The proportion (%) of mutated HIV-Gag CTL epitopes in the reservoir was high and similar in EC and TX (86%[50–100] and 57%[48–82] respectively, p=0.315). Many of them were predicted to negatively impact antigen recognition. Moreover, the proportion of mutated epitopes considered to be CTL-escape variants was also similar in TX and EC (77%[49–92] vs. 50%[33–75] respectively, p=0.117). Thus, the most relevant finding of our study was the high and similar proportions of HIV-Gag CTL-escape mutations in the reservoir of both HIV-noncontroller patients with cART (TX) and patients with spontaneous HIV-control (EC). Our findings suggest that escape mutations of CTL-response may be another obstacle to eliminate the HIV reservoir and constitute a proof of concept that challenges HIV cure strategies focused on the reactivation of reservoirs. Due to the small sample size that could impact the robustness of the study, further studies with larger cohorts of elite controller patients are needed to confirm these results.

## Introduction

Combination antiretroviral therapy (cART) successfully curtails HIV replication and diminishes the circulating virus to undetectable levels [1], improving quality of life of patients with HIV, by decreasing the associated mortality and morbidities [2]. However, cART cannot completely remove HIV and plasma viremia re-appears after 2–8 weeks of treatment discontinuation [3]. Formation of HIV reservoirs within the first few weeks after infection is considered the main barrier to HIV eradication [4]. Because of the latent state of the infected cells that form the HIV reservoir, the expression of viral antigens is silenced. Therefore, virus remains hidden from the immune system.

Among different strategies aimed at eliminating the HIV reservoir, the most studied is the shock and kill strategy [5]. In this strategy, latent HIV can be reactivated by latency reversing agents—LRAs—and eliminated by cART or by the action of the immune system. Although interesting results have been obtained with this strategy, overall its impact on reservoir elimination has been much less than optimal [6,7]. First, HIV reservoir cannot be completely reactivated, with only <1% of replication competent proviruses being reactivated after maximum *in vitro* stimulation [8]. Second, evidence shows that the HIV-specific CD8 (CTL) response of patients starting cART during the chronic phase of infection does not successfully kill infected cells, mainly due to poor antigen recognition of epitopes present in the reservoir, as a result of the accumulation of CTL escape mutations [9].

In this regard, the exceptional group of patients termed elite controllers (ECs), have become the focus of interest as a potential model to attain the so-called functional cure since they can elicit an efficient CTL-mediated immune response to control the virus replication without cART [10]. Given that the status of elite controller is established early from the start of infection [11], our hypothesis is that the reservoir in these patients could be enriched in wild-type sequences of epitopes recognized by CTL cells since the virus has not yet had enough time to select for escape mutations. Thus, CTL response of EC subjects could be effective recognizing their proviruses sequences, and the “shock and kill” strategy could be employed in these patients to eliminate their reservoir.

To test this hypothesis, we studied two different groups of chronic HIV infected subjects with complete suppression of viral replication (elite controllers and patients on cART), and we aimed: (i) to analyse the proportion of mutations in sequences of CTL restricted HIV-Gag epitopes present in the resting memory CD4+ *T*-cells, which is one of the most relevant cellular HIV reservoir [12]; and (ii) to predict the potential impact of these mutations on the patient-specific antigen recognition.

## Materials and methods

### Study population

The two groups of chronically HIV-infected patients with complete viral suppression (<50 copies/mL of plasma HIV-RNA/mL-pVL-) were included in a cross-sectional study: seven elite controller patients (EC group) and 10 patients on successful cART (TX group). EC patients remained with undetectable pVL during at least the last 12 months before their inclusion in the study (determined by at least three consecutive measurements), without receiving cART. TX subjects were defined as patients on cART who also remained with undetectable pVL for at least last 12 months prior to the study. Both groups of patients maintained stable CD4 counts >500 cells/uL. The subjects were recruited at Fundación Jimenez Diaz University Hospital in Madrid, Spain, during a 12-month period. The study protocol was reviewed and approved (Approval ID: PIC-141-2016-FJD) by the Ethical review board (ID: CEIm-Instituto de Investigación Sanitaria-Fundación Jiménez Díaz, Madrid, Spain), in accordance with the declaration of Helsinki. An informed consent form was signed by all patients included in the study.

### Isolation of Trm cells by immunomagnetic technique

Fresh peripheral blood mononuclear cells (PBMCs) were first obtained from 50 mL EDTA-anticoagulated blood samples by density gradient centrifugation with Ficoll-Hypaque (Sigma Chemical Co., USA). The population of resting memory CD4 T (Trm) cells defined by the expression of CD45RO marker (CD45RO+) and lack of expression of activation markers CD69, CD25, and HLADR (CD69-CD25-HLADR-) were purified from totally fresh PBMCs employing immunomagnetic microbeads technology (MACS system, Miltenyi Biotec, Madrid, Spain). Briefly, memory CD4+ *T*-cell subset was first separated by a negative magnetic isolation using the memory CD4 *T*-cell Isolation Kit, following the manufacturer’s instructions. Thereafter, another negative isolation employing monoclonal antibodies against CD25, CD69, and HLA-DR was performed to purify Trm cells. Purity of the isolated cell subset was assessed by flow cytometry (SP6800 Spectral Analyser, Sony, USA). In all the samples, the purity of Trm cell population (expressed as median [Interquartile range]) was 95%[93–97].

### HLA genotyping

Total DNA was extracted employing a QIAamp DNA-MiniKit (Qiagen, USA). HLA class I (A, B, and C) genotyping was performed by polymerase chain reaction sequence-specific oligonucleotide (PCR-SSO) using LIFECODES HLA-SSO kit (Immucor, USA) and subsequent detection with SSO probes of the PCR product by bead array technology (GenProbe, San Diego, CA), with high level of resolution. The acquisition and analysis of the samples was carried out using the Luminex flow analyzer (Millipore, Corporation, Billerica, MA, USA).

### PCR amplification of HIV-Gag gene

Total DNA was obtained from Trm cells employing a QIAamp DNA-MiniKit (Qiagen, USA). Proviral DNA was amplified by nested PCR using the Q5 Hot Start High-Fidelity DNA Polymerase PCR master mix (New England Biolabs, USA). Due to the high genetic variability of HIV-Gag genes, we employed different sets of outer/inner patient-specific primers to successfully amplify the whole gene sequence in all patients (Suplementary Table S1). AMPure XP Kit (Beckman Coulter, USA) and QuantiFluor dsDNA System (Promega, Spain) were used to purify and quantify PCR amplicons respectively.

### Library preparation and Next-Generation Sequencing (NGS)

Approximately 50 ng of DNA fragments of around 400 bp HIV-Gag genes in the autologous virus reservoir of each patient were used to construct DNA libraries with MiSeq Reagent Kit v3 (600-cycle; Illumina Inc, USA), following the manufacturer’s instructions. DNA libraries were pair-end sequenced in a 2 × 300 format using the Illumina MiSeq sequencer (Illumina Inc, USA). The pre-processed reads were subsequently mapped to the H×B2 reference genome (GenBank accession K03455.1) with Bowtie2 (UMIACS, USA). The resulting alignment files were analyzed with VarScan2 software (McDonnell Genome Ins, USA) for variant detection. No large internal deletions were found in the Gag gene of any of the studied subjects. Only variants with at least 20% of frequency along all reads per base were validated. All these programs were accessed using the integrated GPRO suite (Biotechvana, Spain).

### Prediction of optimal HIV-Gag epitopes of CTL immune response

For each subject, optimal HIV-Gag CTL epitopes were determined depending on their HLA class I alleles (A, B, and C), using “the list of the best-defined HIV-1 CTL/CD8+ epitopes” published in the Los Alamos Immunology Database available in September 2019 (www.hiv.lanl.gov/content/immunology/tables/optimal_ctl_summary.html). According to this list, an epitope is considered a best-defined HIV-1 CTL/CD8+ epitope if it meets all of the following criteria: (a) to induce a specific CTL response upon HIV infection *in vivo*; (b) to have the size of an optimal epitope to be recognized at the lowest epitope concentration; and (c) to have identified its HLA class I restricting molecules. To be included in this list, all the best-defined HIV-1 CTL/CD8+ epitopes were tested experimentally and validated mainly by intracellular staining of cytokines (ICS) assay, CD8 Elispot, and/or Chromium-release assays. Autologous sequences of HIV-Gag CTL epitopes of each patient enrolled in the study were checked against the list of the optimal best-defined HIV-1 CTL/CD8+ epitopes from Los Alamos database sequences. The proportion of mutated epitopes, as well as the ratio of mutated amino acids (aas) per epitope (mutated aas/total aas), was calculated for each patient.

### Prediction of CTL escape variants in autologous mutated epitopes

To calculate the proportion of autologous mutated epitopes that could be considered as CTL escape variants the “C*TL/CD8+ Epitope Variants and Escape Mutations* HIV database” (https://www.hiv.lanl.gov/content/immunology/variants/ctl_variant.html) was used. Sequence variants and mutations found in HIV CTL epitopes that have been tested for immunological response are reported in this database. A mutated epitope is considered a CTL escape variant only if it belongs to one of these types of variants: calculated escape; escape documented; inferred escape; literature escape; non-susceptible form; altered epitope processing; or TCR-related mutation.

### Prediction of HLA class I binding for mutated epitopes

The effect of mutated epitopes on recognition by HLA was analyzed employing the latest version of NetMHCpan Server (4.1 version) (DTU Health Tech, University of Denmark), which is the most current and accurate version of the best *in-silico* method to predict the binding strength of HLA molecules with viral peptides by means of an artificial neural network. The next default NetMHC threshold (% rank) values were used to consider the strength of binding of a viral peptide to an HLA allele: strong binding if % rank was ≤0.5, weak binding if % rank was >0.5 to ≤2%), and non-binding if % rank was >2%.

### Statistical analysis

The SPSS software version 15 (SPSS Inc., Chicago, IL, USA) was employed for the statistical analysis of the data. All tests were two-tailed and only *p*-values <0.05 were considered as significant. Due to the relatively small sample size, median [interquartile range] was used to describe the main characteristics of the study population. Non-parametric tests were employed to check for inter-group differences (Chi-squared test for categorical variables and Mann–Whitney *U* test for continuous variables).

## Results

### Characteristics of study population

Clinical characteristics of patients recruited for this study are reported in Table 1. Elite controllers (EC) and treated patients (TX) were similar in age, gender, and CD4 counts. At the moment of inclusion in the study, EC patients had been maintaining the EC status for 5[2–7] years, and TX patients had been on cART for 12[9–16] years. The time since HIV diagnosis was longer for the TX group than for EC; however, viral replication was controlled to undetectable levels in both groups of patients during most of the follow-up. In fact, the levels of CD4 counts were high and quite similar between both groups and, therefore, the level of immunological competence was likely similar in both groups of patients. Protective HLA alleles HLA‐B × 27/B × 57 were not enriched in any particular group of patients (Supplementary Table S2).

**Table 1.** Characteristics of patients included in the study.

Characteristic	Study Group		p-value
	EC	TX	
n	7	10	-
Age (Years)	42 [32-46]	47 [44-51]	0.070
Male (%)	57	90	0.116
CD4 counts (cells/μL)	744 [696-957]	988 [585-1469]	0.526
Time since HIV diagnosis (Years)	5 [4-12]	13 [11-16]	0.011
Lenght of EC status (Years)	5 [2-7]	NA	-
Lenght of treatment (Years)	NA	12 [9-16]	-

Data are expressed as Median [IQR], except sex, expressed as %; *p*-value: comparison between EC and TX groups (Mann-Whitney U test); NA: not apply. EC: elite controller patients; TX: non-controller patients on cART.

### High proportion of mutations in HIV-Gag CTL epitopes in the HIV reservoir of EC and TX patients

**Figure 1.**
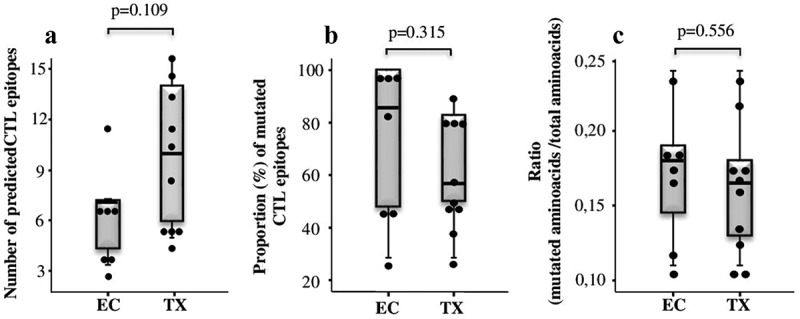
Whisker-Boxplots showing: (a) Number of best-defined CTL epitopes predicted according to the HLA-I haplotype of patients; (b) Proportion (%) of mutated CTL epitopes in autologous sequences obtained from the viral reservoir; (c) ratio of mutated amino acids (mutated amino acids/total amino acids) per individual CTL epitope. *p*-values for the comparisons between EC and TX groups of patients (Mann-Whitney U test) are shown.

We analyzed the proportion of mutations in HIV-Gag CTL epitopes obtained from the viruses archived in the HIV reservoir. For each patient, we first identified (according to the HLA class I haplotype) the best-defined CTL epitopes described in the Los Alamos Immunology Database. The number of optimal HIV-Gag CTL epitopes predicted was similar in EC and TX patients (7 [4–7] and 10 [6–14] respectively; p = 0.109) (Figure 1(a)). Next, we analyzed the entire HIV-Gag sequence of autologous proviral HIV from Trm cells of each patient and identified all mutations in the epitopes of CTL response by comparing with the corresponding sequences of the described optimal CTL epitopes. Surprisingly, the proportion (%) of HIV-Gag CTL epitopes carrying mutations was high in both groups of patients (86% [50–100] in EC and 57% [48–82] in TX, p = 0.315) (Figure 1(b)). Moreover, the ratio of mutated amino acids (mutated amino acids/total amino acids) per individual HIV-Gag CTL epitope was similar in both groups of patients (0.18[0.12–0.19] in EC and 0.16[0.12–0.19] in TX, p = 0.556) (Figure 1(c)).

**Figure 2.**
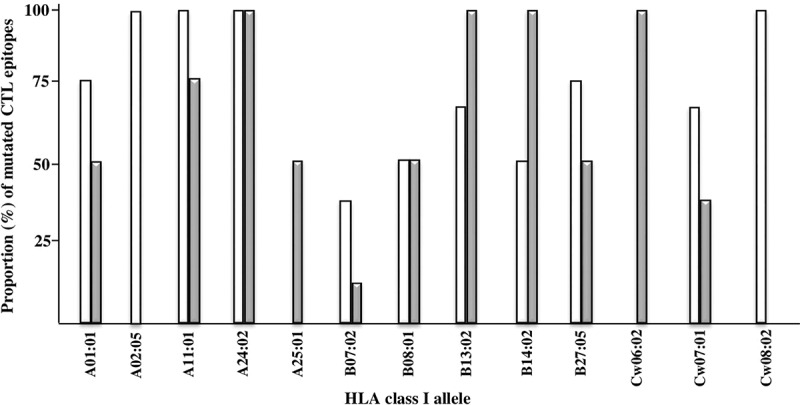
Proportion (%) of autologous mutated CTL epitopes presented by HLA-I alleles that are shared between EC (white bars) and TX (grey bars) groups of patients. No significant differences (chi-square test) were found in the proportion of autologous mutated CTL epitopes between EC and TX groups.

Since the proportion of mutated HIV-Gag CTL epitopes could be influenced by differences in the HLA-I alleles carried by EC and TX patients, we next analyzed the proportion of mutated epitopes on an HLA-specific basis. For this purpose, we selected EC and TX patients sharing a particular HLA-I allele and calculated the proportion of mutated CTL epitopes associated to that allele. Thirteen different HLA-I alleles were shared between EC and TX patients, and the proportion of mutated epitopes associated to these alleles was similar in EC and TX patients (Figure 2).

### Similar proportion of HIV-Gag CTL escape mutations in the viral reservoir of EC and TX patients

**Figure 3.**
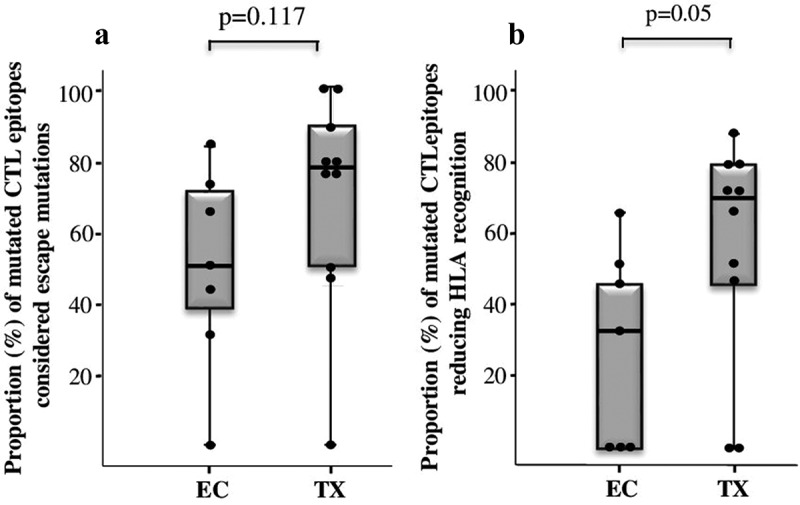
Whisker-Boxplots showing: (a) Proportion (%) of escape mutations within mutated CTL epitopes in EC and TX patients. (b) Proportion (%) of mutated CTL epitopes with a significant impact in reducing HLA recognition in EC and TX patients. *p*-values for the comparison between groups (Mann-Whitney U test) are shown.

Finally, we used the “*CTL/CD8+ Epitope Variants and Escape Mutations*” HIV database to assess the proportion of autologous mutated epitopes in EC and TX patients that had been previously documented as CTL escape variants (www.hiv.lanl.gov/content/immunology/variants/ctl_variant.html). This analysis showed that the frequency of mutated epitopes considered as CTL escape variants were also similar in TX and EC patients (77% [49–92] vs. 50% [33–75] respectively, p = 0.117) (Figure 3(a)).

Additionally, we calculated the potential impact of mutated epitopes on HLA recognition. Interestingly, we found differences between EC and TX patients in the binding affinity to HLA of autologous mutated epitopes (non-binders or weak binder epitopes: 33% [0–50] in EC and 71% [35–80] in TX; p = 0.05) (Figure 3(b)).

## Discussion

To the best of our knowledge, ours is the first study analyzing the CTL escape mutation landscape in HIV-Gag CTL epitopes present in one of the most relevant cell populations (Trm cells) in HIV reservoir, and comparing two groups of HIV patients classified depending on the mechanism of viral replication inhibition: spontaneous (EC group) or cART-mediated (TX group). The results of our study showed that: (a) the proportion of mutated HIV-Gag CTL epitopes present in the viral reservoir was high and similar in EC and in TX patients; (b) a large number of these mutations could potentially affect in a detrimental way the ability of CD8 T cells to recognize the mutated epitopes; c) the proportion of mutated CTL epitopes considered as CTL escape variants was similar in EC and TX patients.

Contrary to expected, our results reveal that EC patients harbor a high frequency of mutations in HIV-Gag CTL epitopes present in the viral reservoir even greater than that found in patients with viral replication suppressed by cART. This high frequency observed in EC was not biased by differences in HLA alleles distribution between groups since the analysis of specific HLA alleles shared between the two groups of patients yielded the same results. Our results agree with previous studies in which immune-escape variants of the CTL response have been observed in EC patients [13–17] even at frequencies similar to patients with uncontrolled viral replication [13]. However, in some of those studies, immune-escape mutations were only analyzed on plasma of EC subjects [13,15]; and no cART-group of patients was evaluated in any of those studies [13–17]. Regarding the studies analyzing mutations present in the HIV reservoir of EC patients, two studies reported the existence of escape mutants in most EC patients, although at low frequencies [14,16]. However, it has to be noted that in both studies the analysis included only epitopes restricted by HLA-B alleles, which could explain the differences in the proportion of mutations in HLA class I-associated epitopes between these studies and our study that included analysis of A, B, and C loci of HLA-I. Interestingly, a more recent study examining a cohort of HIV controllers (including both elite and viremic controllers) also found, in agreement with our results, a high frequency of mutations affecting the interaction with HLA class I molecules, in HIV-Gag epitopes from the viral reservoir [17].

Viral replication control in elite controllers is reached soon after acute infection [11], so the time the virus has to accumulate escape mutations is relatively short, and thus it does not seem that such a high number of mutations can be generated during this short period of time. Nevertheless, several mechanisms are likely involved in the accumulation of mutations in CTLs epitopes observed in EC patients: first, the strong selection pressure exerted by an efficient CTL response in EC may contribute to the rapid accumulation of mutations in epitopes of CTL response [18]. Second, some of the mutations found could be transmitted by the infecting viral strain rather than being the consequence of escape in the new host, as has been previously suggested [19]. Third, productive HIV replication at low levels under the limit of detectability by commercial assays observed in EC patients [20]; could lead to mutations that can be accumulated in the reservoir over the course of the viral cycle.

The most relevant finding of our study was a similar proportion of HIV-Gag CTL escape mutations in the viral reservoir of EC and TX patients. The majority of mutated variants in non-controller patients on cART had reduced affinity for HLA molecules and were included as escape variants in the “*CTL/CD8+ Epitope Variants and Escape Mutations*” database, suggesting that these mutated variants could escape CTL recognition. In agreement with this, a previous study has shown a high prevalence of escape mutants to the CTL response in the reservoir of non-controller patients on cART [9]. Surprisingly, half of the mutated variants in EC patients were also included as CTL escape variants, although most of them conserved a strong binding affinity to HLA-I molecules, which suggests that EC patients may retain the ability to recognize epitope variants that arise as a result of immune selection pressure, as has been proposed by Liu et al., 2006 [21]. In agreement with this, previous studies had shown that elite controllers can develop de novo CTL responses able to recognize and suppress these variants [13–15,22].

Some limitations of our study deserve to be commented on. First, the sample size analyzed limits the robustness of the conclusions and thus further studies with large cohorts of patients are needed to support our findings. Second, we employed a set of optimally-defined dominant CTL epitopes and thus we lacked information about subdominant epitopes that may be relevant in the CTL-mediated control of HIV replication as suggested by Frahm et al., 2006 [23]. Finally, some of our results are derived from *in silico* studies, and thus, it is of utmost importance to corroborate them with studies employing functional assays to measure CTL response to viral variants in these patients.

Taken together, our results reveal a high frequency of CTL mutations both in the HIV reservoir of non-controller subjects with successful cART-mediated control and in the HIV reservoir of subjects that maintain spontaneous HIV-control since an early stage of infection. In fact, a large number of these mutations could potentially affect in a detrimental way the process of antigen recognition by CD8 T cells and have been included as CTL escape variants. Our findings suggest that escape mutations of CTL response may be another obstacle to eliminate the HIV reservoir and constitute a proof of concept that challenges HIV cure strategies focused on the reactivation of viral reservoirs.

## Supplementary Material

Supplemental Material

## Data Availability

Data that support the findings of this study are available from the corresponding author upon reasonable request.
