# Supplementary material for: High frequency of CD8 escape mutations in elite controllers as new obstacle for HIV cure
Source: Virulence. 2022 Oct 3;13(1):1713–9. doi: 10.1080/21505594.2022.2129353 (PMC9543107; doi:10.1080/21505594.2022.2129353)
Supplement: Supplemental Material [file KVIR_A_2129353_SM5759.zip › supplementary/Supplementary Table 1.docx]

**Table 1**. Characteristics of patients included in the study.

| **Characteristic** | **Study Group** | | **p-value** |
| --- | --- | --- | --- |
|  | **EC** | **TX** |  |
| **n** | 7 | 10 | - |
| **Age (Years)** | 42 [32-46] | 47 [44-51] | 0.070 |
| **Male (%)** | 57 | 90 | 0.116 |
| **CD4 counts (cells/μL)** | 744 [696-957] | 988 [585-1469] | 0.526 |
| **Time since HIV diagnosis (Years)** | 5 [4-12] | 13 [11-16] | 0.011 |
| **Lenght of EC status (Years)** | 5 [2-7] | NA | - |
| **Lenght of treatment (Years)** | NA | 12 [9-16] | - |

Data are expressed as Median [IQR], except sex, expressed as %; p-value: comparison between EC and TX groups (Mann-Whitney U test); NA: not apply. EC: elite controller patients; TX: non-controller patients on cART.
