# Supplementary material for: High frequency of CD8 escape mutations in elite controllers as new obstacle for HIV cure
Source: Virulence. 2022 Oct 3;13(1):1713–9. doi: 10.1080/21505594.2022.2129353 (PMC9543107; doi:10.1080/21505594.2022.2129353)
Supplement: Supplemental Material [file KVIR_A_2129353_SM5759.zip › supplementary/Supplementary Table 2.docx]

**Supplementary Table 2**. HLA-I alleles of patients included in the study.

| Group of patient | Alleles HLA-I A | | Alleles HLA-I B | | Alleles HLA-I C | |
| --- | --- | --- | --- | --- | --- | --- |
| EC | 02:05 | 25:01 | 07:02 | 14:02 | 07:02 | 08:02 |
| EC | 30:02 | 33:01 | 39:10 | 58:01 | 03:02 | 12:03 |
| EC | 02:05 | 24:02 | 27:05 | 58:01 | 02:02 | 07:01 |
| EC | 01:01 | 33:01 | 14:02 | 58:01 | 07:01 | 08:02 |
| EC | 02:02 | 11:01 | 45:01 | 45:01 | 16:01 | 16:01 |
| EC | 01:01 | 11:01 | 08:01 | 27:05 | 01:02 | 07:01 |
| EC | 01:01 | 02:01 | 40:01 | 57:01 | 06:02 | 07:01 |
| TX | 02:01 | 11:01 | 07:02 | 40:02 | 02:02 | 07:02 |
| TX | 03:01 | 03:01 | 07:02 | 14:02 | 07:02 | 15:05 |
| TX | 25:01 | 33:01 | 15:01 | 53:01 | 03:03 | 04:01 |
| TX | 03:01 | 03:01 | 27:05 | 51:01 | 02:02 | 14:02 |
| TX | 02:01 | 02:01 | 13:02 | 52:01 | 06:02 | 12:02 |
| TX | 02:01 | 02:01 | 18:01 | 39:06 | 07:01 | 07:02 |
| TX | 02:05 | 24:02 | 07:02 | 14:02 | 07:02 | 08:02 |
| TX | 01:01 | 68:02 | 08:01 | 27:05 | 02:02 | 07:01 |
| TX | 29:02 | 68:02 | 35:08 | 53:01 | 04:01 | 04:01 |
| TX | 11:01 | 23:01 | 15:17 | 39:01 | 07:01 | 12:03 |

Data obtained from HLA genotyping of each patient. EC: elite controller patients; TX: patients on successful cART
